# Efficacy and Safety of Sitafloxacin in the Treatment of Acute Bacterial Infection: A Meta-analysis of Randomized Controlled Trials

**DOI:** 10.3390/antibiotics9030106

**Published:** 2020-03-02

**Authors:** Chao-Kun Chen, I-Ling Cheng, Yu-Hung Chen, Chih-Cheng Lai

**Affiliations:** 1 Department of Surgery, Chi Mei Medical Center, Tainan 73657, Taiwan; a.kun.ke@gmail.com; 2 Department of Pharmacy, Chi Mei Medical Center, Liouying, Tainan 73657, Taiwan; bokey1010@gmail.com (I.-L.C.); her0windqoo@gmail.com (Y.-H.C.); 3 Department of Internal Medicine, Kaohsiung Veterans General Hospital, Tainan Branch, Tainan 73657, Taiwan

**Keywords:** sitafloxacin, acute bacterial infection, complicated urinary tract infection, acute pyelonephritis, pneumonia

## Abstract

This meta-analysis aimed to assess the efficacy and safety of sitafloxacin in treating acute bacterial infection. PubMed, Embase, and Cochrane databases were searched up to August 13, 2019. Only randomized controlled trials (RCTs) evaluating sitafloxacin and comparators in the treatment of acute bacterial infections were included. The outcomes were clinical and microbiological responses and the risk of adverse event (AE). Five RCTs were enrolled, including 375 and 381 patients who received sitafloxacin and the comparator, respectively. Overall, the clinical response rate of sitafloxacin in the treatment of acute bacterial infections was 94.6%, which was noninferior to that of the comparator (92.5%) (odds ratio (OR), 1.01; 95% CI, 0.24–4.32; *I*^2^ = 66%). For patients with complicated urinary tract infection (cUTI)/acute pyelonephritis (APN), the clinical response rate of sitafloxacin and the comparator was 96.9% and 91.3%, respectively (OR, 2.08; 95% CI, 0.35–12.44; *I*^2^ = 54%). For patients with pneumonia, the clinical response rate of sitafloxacin was 88.6%, which was comparable to that of the comparator (OR, 0.36; 95% CI, 0.11–1.21; *I*^2^ = 0%). The microbiological response of sitafloxacin was 82.0%, which was noninferior to that of the comparator (77.8%) (OR, 1.59; 95% CI, 0.77–3.28; *I*^2^ = 47%). The risk of treatment-emergent adverse event (TEAE), drug-related TEAE, and all-cause mortality were similar between sitafloxacin and the comparators (TEAE, OR, 1.14; 95% CI, 0.64–2.01, drug-related TEAE, OR, 1.14; 95% CI, 0.48–2.69, mortality, OR, 0.93; 95% CI, 0.09–9.44). In conclusion, sitafloxacin is noninferior to other commonly used antibiotics with respect to both clinical and microbiological response rates in patients with an acute bacterial infection, including cUTI/APN and pneumonia. In addition, sitafloxacin is also as safe as the comparators.

## 1. Introduction

Sitafloxacin is a new generation fluoroquinolone that exhibits excellent in vitro activity against many Gram-positive, Gram-negative, anaerobic bacteria, and atypical pathogens. Moreover, it remains active against the strains resistant to other fluoroquinolones [1]. For commonly encountered bacteria, the activity of sitafloxacin against Gram-positive cocci, including *Streptococcus pneumoniae*, *Streptococcus pyogenes*, *Enterococcus faecalis*, and methicillin-susceptible *Staphylococcus aureus* was comparable or superior to those of garenoxacin, moxifloxacin and levofloxacin [2]. In addition, sitafloxcain showed the more potent activity against Gram-negative bacteria, including *Escherichia coli*, *Hemophilus influenzae*, *Moraxella catarrhalis*, *Enterobacteriaceae*, and *Pseudomonas aeruginosa* and anaerobic bacteria other than fluoroquinolones—garenoxacin, moxifloxacin, and levofloxacin [2]. For atypical bacteria, the minimum inhibitory concentration (MIC) of sitafloxacin at which 90% of isolates (MIC₉₀) against *Mycoplasma pneumoniae*, was 0.03 μg/mL, which was 4- and 16-fold more active than moxifloxacin and levofloxacin, respectively [3]. MIC₉₀ of sitafloxacin against *Legionella pneumophila* was 0.004 μg/mL, which was 2- and 4-fold more active than levofloxacin and moxifloxacin, respectively [3]. Even for a multidrug resistant organism, such as the carbapenem-resistant *Acinetobacter baumannii* complex, sitafloxacin had a significantly lower MIC in comparison with ciprofloxacin and levofloxacin, and the rate of resistance to sitafloxacin was significantly lower than that to ciprofloxacin and levofloxacin [4]. All these findings indicate that sitafloxacin has great activity against these commonly encountered pathogens in the clinical entity of respiratory tract infection and urinary tract infection and further suggests that sitafloxacin could be a promising antibiotic in the treatment of acute bacterial infection. 

Sitafloxacin has been used in the treatment of respiratory tract infection and urinary tract infection in Japan for decades, and it has become available in Thailand since 2012. However, only limited studies have investigated the clinical efficacy of sitafloxacin in the treatment of pneumonia and urinary tract infections [5,6,7,8,9,10,11]. To provide better evidence of the efficacy and safety of sitafloxacin on treating acute bacterial infections, we conducted this comprehensive and updated meta-analysis. 

## 2. Methods

### 2.1. Study Search and Selection

A systematic review of the literature in PubMed, Embase, and Cochrane databases was conducted using the following search terms: “sitafloxacin,” “randomized,” and “randomised”, until August 13, 2019. We only included randomized controlled trials (RCT) that investigated the clinical efficacy and safety of sitafloxacin and other comparators for treating acute bacterial infections. The single-arm study, case series, or cohort studies, pharmacokinetic studies, or in vitro studies and studies focusing on drug toxicity were excluded. Authorship, publication year, study sites, antibiotic regimens, clinical and microbiological outcomes, and adverse events (AEs) were extracted from the included studies.

### 2.2. Definition and Outcome

The intention-to-treat (ITT) population was defined as subjects who had acute bacterial infection according to the inclusion criteria for clinical trial and received any amount of the study drug. The clinically evaluable (CE) population was defined as the ITT population who had an available outcome assessment. The microbiologically evaluable (ME) population was defined as the CE population, in which at least one bacterial pathogen was isolated at baseline.

The primary outcome was measured as the clinical response at the end of treatment (EOT) and test of cure (TOC) visit among the ITT and the CE population. Clinical response was defined as the resolution or improvement of clinical signs and symptoms of acute bacterial infection, and no further antimicrobial therapy was needed. We also measured microbiological response and the risk of AEs as secondary outcomes.

### 2.3. Data Analysis

This risk of bias of enrolled RCTs was evaluated by the Cochrane Risk of Bias Assessment Tool [12]. We used the random effect model of the software Review Manager, version 5.3, to conduct statistical analyses. The outcome analysis was calculated using the pooled odds ratio (OR) and 95% confidence intervals (CIs).

## 3. Results

### 3.1. Study Selection and Characteristics

**Figure 1:**
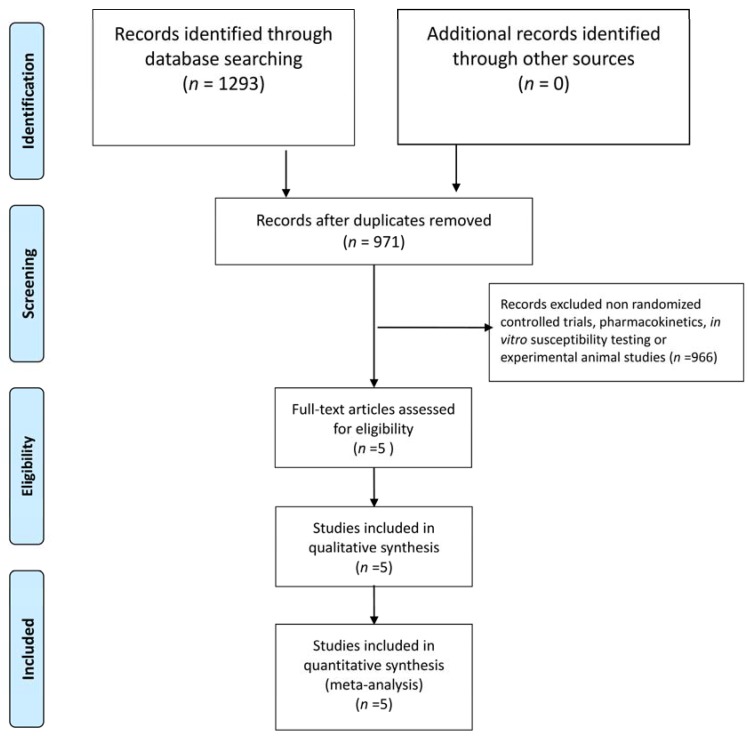
The flow-chart of study selection.

**Figure 2:**
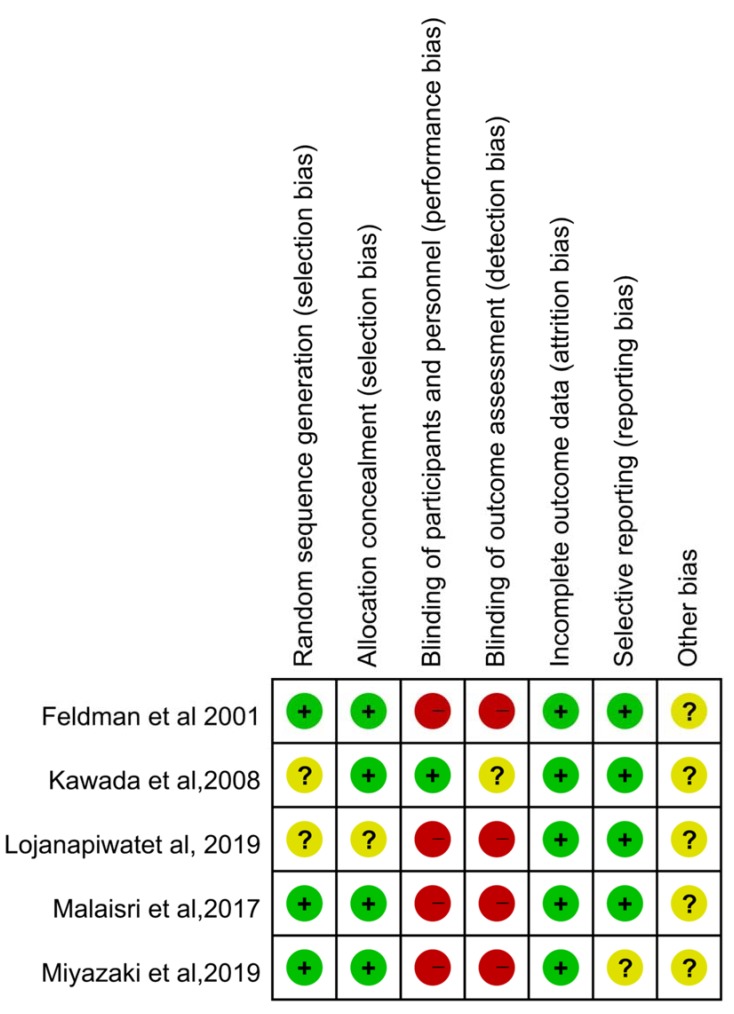
The summary of risk of bias.

The initial literature search identified 1293 studies from PubMed (n = 317), Embase (n = 912), and the Cochrane database (n = 64). After excluding 322 duplicated articles, the remaining 971 articles were screened using the title and abstract. Finally, a total of five studies were enrolled after full-text screening (Figure 1). Among them, four studies carried a high risk of performance and detection bias (Figure 2). In total, five RCTs [5,7,8,9,11] fulfilling the inclusion criteria were enrolled in this meta-analysis (Table 1) [5,7,8,9,11]. Except for one study [8], all the others [5,7,9,11] were multicenter studies. Three studies [7,8,11] focused on complicated urinary tract infection (cUTI)/acute pyelonephritis (APN), and two [5,9] focused on pneumonia. Overall, each of the 375 and 381 patients received sitafloxacin and the comparator, respectively. One study [5] used sitafloxacin in the intravenous form, and four studies [7,8,9,11] used sitafloxacin in the oral form. However, the dosage of sitafloxacin varied and ranged from 100 mg per day to 400 mg per day. The comparative agents also varied in each study and included imipenem [5], ertapenem [8], levofloxacin [11], garenoxacin [9], and ceftriaxone/cefdinir [7].

### 3.2. Clinical Efficacy

**Figure 3:**
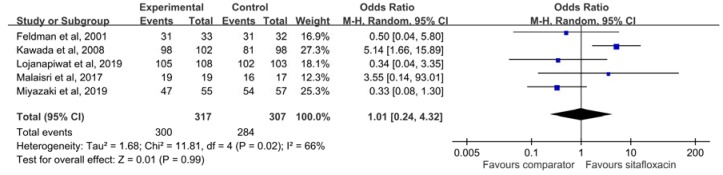
The clinical response rate of sitafloxacin and the comparator in the treatment of acute bacterial infections at the end of treatment visit among the clinical evaluable population.

The clinical response rate of sitafloxacin in the treatment of acute bacterial infections at EOT among the CE population was 94.6%, which was similar to that of the comparator (92.5%) (OR, 1.01; 95% CI, 0.24–4.32; *I*^2^ = 66%) (Figure 3). The similarity did not differ in the sensitivity analysis. These findings did not change according to oral form (OR, 1.16; 95% CI, 0.21–6.52; *I*^2^ = 73%) or intravenous form (OR, 0.50; 95% CI, 0.04–5.80). The clinical failure rate at EOT of sitafloxacin was only 4.1%, which was similar to that of the comparator (7.5%) (OR, 0.75; 95% CI, 0.21–2.65; *I*^2^ = 52%). The similarity between sitafloxacin and the comparator was also observed in oral form (OR, 0.73; 95% CI, 0.16–3.32; *I*^2^ = 63%) or intravenous form (OR, 0.97; 95% CI, 0.06–16.18). Among the ITT population, no significant difference was observed regarding the clinical response rate at EOT (88.7% vs. 86.0%, OR, 1.26; 95% CI, 0.69–2.28; *I*^2^ = 0%). The clinical response rate and clinical failure rate at TOC among the CE population was similar between sitafloxacin and the comparator (clinical response rate: 95.3% vs. 93.8%, OR, 0.98; 95% CI, 0.41–2.34; *I*^2^ = 0%, and clinical failure rate: 4.2% vs. 4.4%, OR, 1.00; 95% CI, 0.40–2.49; *I*^2^ = 0%).

In the subgroup analysis of cUTI/APN based on three studies [7,8,11] the clinical response rate of sitafloxacin and the comparator was 96.9% and 91.3%, respectively, and no significant difference between them was observed (OR, 2.08; 95% CI, 0.35–12.44; *I*^2^ = 54%). For patients with APN in three studies [7,8,11], sitafloxacin exhibited a similar clinical response rate with the comparator (OR, 1.90; 95% CI, 0.46–7.83; *I*^2^ = 0%). In the subgroup analysis of pneumonia, the pooled analysis of the two studies [5,9] showed that the clinical response rate of sitafloxacin was 88.6%, which was comparable to that of the comparator (OR, 0.36; 95% CI, 0.11–1.21; *I*^2^ = 0%).

### 3.3. Microbiological Response 

**Figure 4:**
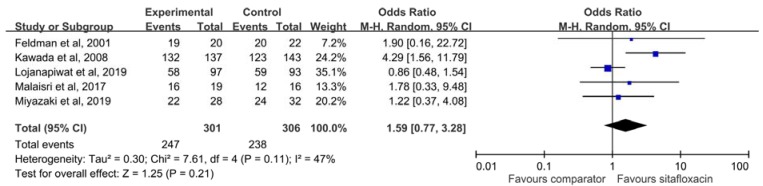
The microbiological response rate of sitafloxacin and the comparator for treating acute bacterial infections.

Overall, the microbiological response of sitafloxacin in the treatment of acute bacterial infection was 82.0%, which was similar to that of the comparator (77.8%) (OR, 1.59; 95% CI, 0.77–3.28; *I*^2^ = 47%) (Figure 4). This finding did not change according to oral form (OR, 1.59; 95% CI, 0.70–3.62; *I*^2^ = 60%) or intravenous form (OR, 1.90; 95% CI, 0.16–22.72). Subgroup analysis did not find significant difference between sitafloxacin and the comparator for patients with cUTI/APN (OR, 1.77; 95% CI, 0.57–5.56; *I*^2^ = 73%) and pneumonia (OR, 1.33; 95% CI, 0.45–3.93; *I*^2^ = 0%). 

### 3.4. Risk of Adverse Event

Four studies [5,7,9,11] reported the risk of treatment-emergent adverse event (TEAE), and we found the risk of TEAE was similar between sitafloxacin and the comparator (OR, 1.14; 95% CI, 0.64–2.01; *I*^2^ = 61%). Three studies [5,7,9] reported the risk of drug-related TEAE, and the risk of sitafloxacin-related TEAE was similar to that of the comparator (14.4% vs. 12.9%, OR, 1.14; 95% CI, 0.48–2.69; *I*^2^ = 56%). In the pooled analysis of three studies [5,7,8] reported mortality, the all-cause mortality of sitafloxacin was 0.49%, which was as low as that of the comparator (0.49%) (OR, 0.93; 95% CI, 0.09–9.44; *I*^2^ = 2%).

## 4. Discussion

This meta-analysis investigating the use of sitafloxacin in the treatment of acute bacterial infections has several significant findings. First, sitafloxacin exhibited a similar clinical response and failure rate with the comparators at EOT among the CE population. Second, the similarity between sitafloxacin and the comparator was also revealed in many ways, such as the clinical efficacy at the TOC visit, clinical response rate among the ITT population, and sensitivity analysis. Third, the subgroup analysis demonstrated that the clinical response rates of sitafloxacin in the treatment of cUTI/APN, only APN, and pneumonia were comparable to the comparators. Finally, the microbiological response rate between sitafloxacin and the comparator was similar in the pooled analysis of five RCTs and even the subgroup analysis of cUTI/APN and the pneumonia group. Therefore, these findings suggest that the clinical efficacy of sitafloxacin is comparable to other antibiotics in the treatment of acute bacterial infection.

In addition to these clinical findings, four enrolled studies [7,8,9,11] in this meta-analysis demonstrated the sitafloxacin exhibited potent in vitro activity. In the Lojanapiwat et al. study [7], the antibiotic-resistant rate of sitafloxacin was only 5.9% (17/289), which was lower than the comparators (ceftriaxone: 10.7%, and cefdinir, 12.1%). In the Kawada et al study [11], the MIC_50_ and MIC_90_ of sitafloxacin was lower than levofloxacin (MIC_50_: 0.1 μg/mL vs. 0.78 μg/mL, MIC_90_: 1.56 μg/mL vs. ≥ 25 μg/mL). In the Miyazaki et al. study [9], the MICs of sitafloxacin against most of the Gram-negative bacteria were lower than those of garenoxacin. Even for ESBL-producing pathogens, the sitafloxacin resistant rate was only 5.6% (2/36) in Malasiri et al.’s study [8]. All these findings were consistent with previous studies [4,13,14,15,16] and confirmed the potent in vitro activity of sitafloxacin. Therefore, the favorable clinical efficacy of sitafloxacin in the treatment of acute bacterial infections may be partially explained by the findings of these in vitro studies.

We also found that the risk of AE among patients receiving sitafloxacin was as low as other comparative agents in this meta-analysis. The risks of TEAE, drug-related TEAE, and all-cause mortality did not differ between sitafloxacin and the comparators. Therefore, it should suggest that sitafloxacin was as tolerable as the comparator in the clinical uses.

This meta-analysis has several limitations. First, most of the included studies were conducted in Asia, particularly Japan and Thailand, and the number of enrolled patients and study were limited. Therefore, the finding of this meta-analysis may not be generalized to Western countries. Second, the dosage of sitafloxacin varied in different studies, which may affect the clinical efficacy and safety of this novel agent. Third, only two types of infections—cUTI/APN and pneumonia were included in this meta-analysis. Although these two types of infections are the most common type of infection in the real world, we still need further studies to explore the use of sitafloxacin in other types of infections.

In conclusion, sitafloxacin is noninferior to other comparators in both clinical and microbiological response rates for treating patients with an acute bacterial infection. Furthermore, sitafloxacin is as tolerable as the comparators.

## Figures and Tables

**Table 1:** Summary of the characteristics of enrolled studies in the meta-analysis.

Study, Published Year	Study Design	Study Site	Study Period	Study Population	No of Patients (ITT Population)		Dose regimen	
					Sitafloxacin	Comparator	Sitafloxacin	Comparator
Feldman et al., 2001	phase II, randomized, open-label, parallel trial	10 centers in three countries	NA	Acute bacterial pneumonia requiring hospitalization	35	34	Intravenous sitafloxacin 400 mg qd	Imipenem 500 mg q8h
Kawada et al., 2008	randomized double-blind trial	58 hospitals in Japan	1998–2000	Complicated urinary tract infection	122	121	Oral sitafloxacin 50 mg b.i.d	Oral levofloxacin 100 mg t.i.d
Malaisri et al., 2017	prospective, open-label, randomized, controlled trial	Single hospital in Thailand	2012–2015	Acute pyelonephritis caused by ESBL *E. coli*	19	17	Oral sitafloxacin 100 mg bid followed by initial 3-day carbapenem	Intravenous ertapenem 1 g qd followed by initial 3-day carbapenem
Lojanapiwat et al., 2019	prospective, open-label, randomized, controlled, noninferiority, clinical trial	9 medical centers in Thailand	2013 to 2015^9^	Acute pyelonephritis or complicated urinary tract infection	141	148	Oral sitafloxacin 100 mg bid	Intravenous ceftriaxone 2 g qd x 2–3 days, followed by oral cefdinir 100 mg q8h
Miyazaki et al., 2019	randomized, open-label clinical trial	11 medical centers or hospitals in Japan	2013–2017	Elderly patients with pneumonia	58	61	Oral sitafloxacin 100 mg qd	Oral garenoxacin 400 mg qd
